# Supplementary material for: Learning curve and surgical outcome of robotic assisted colorectal surgery with ERAS program
Source: Sci Rep. 2022 Nov 29;12:20566. doi: 10.1038/s41598-022-24665-w (PMC9709162; doi:10.1038/s41598-022-24665-w)
Supplement: Supplementary file 1 — Supplementary Information 1. [file 41598_2022_24665_MOESM1_ESM.zip › LSC Raw data-Ñ[▒K/116/N_ñw╝╨░O▒Kñσ.pdf]

W65 / W65

臺中榮民總醫院
手術室護理記錄

[redacted]

基本資料

- 室別： 開刀房-18室，手術科別： CRS，手術類別： 常規，日期： 2021/02/05
- 進入時間： 1100 ； 麻醉開始時間： 1110 ； Time Out時間： 1159 ； 劃刀時間： 1200 ； 手術結束時間： 1725 送出時間： 1745 ；
- 麻醉方式： GE
  診斷：A COLON CA WITH LIVER METS
  手術名稱：Laparoscopic Anterior resection and anastomosis - malignant
  手術局部用藥：nil
  術前預防性抗生素：
  - 抗生素名稱一： Veterin 1GM IVD由麻醉護理師給予
  - 時間： 1140
  - 上次簽章: 朱芸萱

  ，追加預防性抗生素：
  - 抗生素名稱一： Veterin 1GM IVD由麻醉護理師給予
  - 時間： 1540
  - 上次簽章: 陳祐婷
- 手術醫師： 蔣鋒帆，陳映菁，林志安，黃偲霖
- 麻醉醫師： VS 張思玲
-
  - 刷手護士A ： 林怡廷，時間： 1100，接替刷手一： 朱芸萱，時間： 1230，接替刷手二： 林愛甯，時間： 1500
  - 流動護士A ： 朱芸萱，時間： 1100，接替流動一： 陳心慈，時間： 1200，接替流動二： 林怡廷，時間： 1300
- 更多接替及流動護士： 接替流動三： 陳祐婷，時間： 1500

皮膚狀況

A-V Shunt： 無
術前： 完整
記錄者： 上次簽章: 朱芸萱
術後： 完整
記錄者： 上次簽章: 陳祐婷

手術準備

手術臥式 單一手術臥式

-
  - 手術臥式： 截石臥式
  - 使用附件： 溫毯 43 ℃，頭圈，腳架
  - 皮膚準備： 10%Aq Betadine，Hibiscrub，2% Chlorhexidine
  - 電燒： 使用電燒: 電極片位置： 右大腿，記錄者： 上次簽章: 朱芸萱

止血帶T： 無

植入物

- 植入物名稱/號碼/數量/部位：ETHICON ENDO GIA 60-4.1mm*3/A-colon
  EEA 33mm*1/腸斷端
  防沾黏(小)*1/腹腔

，記錄者： 上次簽章: 林怡廷

標本送檢

- 細菌培養 無
- 細胞學 無
- 冰凍切片 無
- 病理標本

組織名稱一： Rectum
,
組織名稱二： IMA Root
,
組織名稱三： Distal cut end
, 更多組織名稱
    - 組織名稱四： Peritoneum tumor
- 截肢 無

標本交付 標本： 3 袋 , 標本： 0 盒 , 標本： 1 瓶 , 細胞學： 0 管 , Culture Tube： 0 管 , 被交付醫師： R 林志安 ,

- 記錄者： 上次簽章: 陳祐嫭
- 時間： 1700

傷口術後敷料

傷口敷料： 傷口敷料： 無

- 請於圖上標示部位：

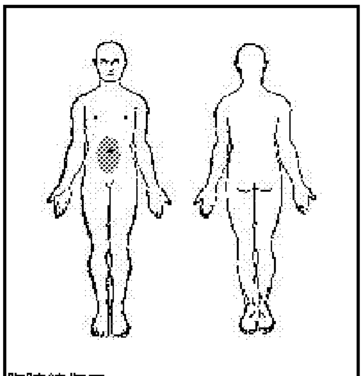

- 傷口敷料： 軟性敷料

導管

導管	天數	導管資訊	插入日期	插入時間	更換日期	拔除日期	拔除時間	拔除原因	預計拔除/更換	預計拔除/更換日期
A-line (1)	1	號碼:20G ; 部位:右手;	2021/02/05	1120						
Penrose Drain(1)	1	號碼:12 mm.; 部位:骨盆腔;	2021/02/05	1520						
Sump NG Tube(1)	1	號碼:16 Fr.; 深度:65cm; 部位:胃;	2021/02/05	1130						
Foley(1)	1	號碼:16 Fr.; 部位:膀胱;	2021/02/05	1135						

用物計數

- 手術中出血量： 100 c.c.
- 手術中尿量： 650 c.c.
-
    - 其它： 無

用物計數： 多切口同時縫合

- 1.器械： 正確 , 2.縫針： 正確 , 3.刀片： 正確 , 4.紗布： 正確 , 5.腹部墊： 未使用 , 6.紗球： 未使用 , 7.PEANUTS ： 未使用 , 8.紗條： 未使用 , 9.絲帶： 未使用 , 10.針頭： 未使用 , 11.Vessel loop： 未使用 , 12.Boots： 未使用 , 13.Jelco： 未使用 , 14.棉條： 未使用 ,
    - 第一次：
        - 刷手護士 ： 林愛甯
        - 流動護士： 上次簽章: 陳祐嫭
        - 時間： 1540

,
    - 第二次：
        - 刷手護士 ： 林愛甯
        - 流動護士： 上次簽章: 陳祐嫭

        - 時間： 1550

,

- 第三次：
    - 刷手護士 ： 林愛甯
    - 流動護士： 上次簽章: 陳祐嫥
    - 時間： 1600

備註

備註：R6 olympus scope NO.2。

- 記錄者： 上次簽章: 朱芸萱
- 時間： 1100

填寫護理人員 陳祐嫥

正式摘要

W65 / W65

doctorRank:B
